# Some long-term effects of scoliosis diagnosed at school age

**DOI:** 10.1186/1748-7161-7-S1-O8

**Published:** 2012-01-27

**Authors:** O Nowotny-Czupryna, A Kowalczyk, K Czupryna, J Nowotny, M Plaszewski

**Affiliations:** 1 Medical University of Silesia in Katowice Katowice, Poland; 2 Institute of Physiotherapy – Higher School of Administration in Bielsko-Biała, Poland

## Background

Abnormal arrangement of the body may lead to the development of overload syndrome, nerve roots irritation, pain, ventilator impairment and also worsening of physical fitness [1-3].

## Objective

Checking how, after years low degree scoliosis impairs breathing, reduces performance and generates back pain was the aim of the study.

## Materials and methods

Respiratory function, working capacity (PWC170) and pain intensity (Jackson and Moskowitz regimen) were assessed in 39 adults, aged from 19 to 38 years, who were diagnosed in adolescence with low degree scoliosis (10°-28°). Also, 43 controls with no scoliosis in adolescence were examined.

## Results

There was no progression of the curvature after the treatment. Spirometric results among scoliotic subjects were slightly lower than in the controls, although it did not show the characteristics of the restrictive type of respiratory disorder, which was found in 5.1% of patients. PWC170 test results were significantly lower (by about 20%) than in controls, and 84.6% subjects reported periodical, occasional of frequent, mostly lumbar pain associated with the work performed. In 12.8% cases pain impeded breathing. In about half of the group pain occurred especially after physical effort and caused limitation of activity, while in other subjects did not affect daily activities.

## Conclusions

1) subjects with low degree scoliosis generally do not indicate impairment of lung ventilation with the characteristics of restrictive disorders; 2) adults with settled low degree scoliosis characterized by lack of physical fitness, in the form of reductions in PWC170; 3) the presence of school-age low degree scoliosis predisposes to the occurrence of pain symptoms in adulthood.
